# CYP2E1 RsaI/PstI Polymorphism and Gastric Cancer Susceptibility: Meta-Analyses Based on 24 Case-Control Studies

**DOI:** 10.1371/journal.pone.0048265

**Published:** 2012-11-05

**Authors:** Wenlei Zhuo, Liang Zhang, Yan Wang, Junjun Ling, Bo Zhu, Zhengtang Chen

**Affiliations:** 1 Institute of Cancer, Xinqiao Hospital, Third Military Medical University, Chongqing, China; 2 Department of Environmental Hygiene, College of Preventive Medicine, Third Military Medical University, Chongqing, China; 3 Institute of Respiratory Diseases, Xinqiao Hospital, Third Military Medical University, Chongqing, China; 4 Department of Otolaryngology, Southwest Hospital, Third Military Medical University, Chongqing, China; National Cancer Center, Japan

## Abstract

**Background:**

Previous reports implicate CYP2E1 RsaI/PstI polymorphism as a possible risk factor for several cancers. Published studies on the relationship of CYP2E1 RsaI/PstI polymorphisms with the susceptibility to gastric cancer are controversial. This study aimed to determine this relationship accurately.

**Methods:**

Meta-analyses that assessed the association of CYP2E1 RsaI/PstI variations with gastric cancer were conducted. Subgroup analyses on ethnicity, smoking status, alcohol consumption, and source of controls were also performed. Eligible studies up to Mar 2012 were identified.

**Results:**

After rigorous searching and screening, 24 case-control studies comprising 3022 cases and 4635 controls were selected for analysis. The overall data failed to indicate the significant associations of CYP2E1 RsaI/PstI polymorphisms with the gastric cancer risk [c2 vs. c1: odds ratio (OR) = 1.06; 95% confidence interval (CI) = 0.88–1.28; c2c2 vs. c1c1: OR = 1.23; 95% CI = 0.78–1.92; c2c2+c1c2 vs. c1c1: OR = 0.93; 95% CI = 0.79–1.10]. Similar results were observed in the subgroup analyses on ethnicity, drinking status, and source of controls. However, in the subgroup analysis on smoking status, a borderline increase in cancer risk was found among long-term smokers (c2c2+c1c2 vs. c1c1: OR = 1.39; 95% CI = 1.00–1.92).

**Conclusion:**

CYP2E1 RsaI/PstI polymorphisms may modify the susceptibility to gastric cancer among individuals who have a smoking history. Large and well-designed studies are needed to confirm this conclusion.

## Introduction

Gastric cancer is one of the most common cancers in the world, accounting for 8% of the total cancer cases and resulting in 10% of the total deaths. Over 70% of new cases and deaths occur in developing countries [1]. The mechanisms of gastric carcinogenesis are still unknown. Previous epidemiological investigations indicate that smoking, drinking, and *Helicobacter pylori* infection may be risk factors for gastric cancer [2], [3]. Nevertheless, only a small proportion of the people exposed to these environmental factors eventually develop gastric cancer, indicating that host genetic factors may have critical functions in gastric carcinogenesis. Therefore, the interactions of genetic factors with environmental factors may contribute to increased gastric carcinoma susceptibility [4].

Only a few gene polymorphisms associated with gastric cancer risk have been identified. Metabolizing enzymes are involved in the bioactivation and detoxification of xenobiotics. Cytochrome P4502E1 (CYP2E1), a member of the cytochrome P450 superfamily, is an ethanol-inducible enzyme that metabolically activates various carcinogens, such as benzene, vinyl chloride, and N-dimethylnitrosamines [5], [6]. The activation of nitrosamines is believed to be related to the development of various cancers [7]. Several single nucleotide polymorphisms in CYP2E1 gene have been identified. RsaI/PstI polymorphisms, which are in complete linkage disequilibrium, in the 5′-flanking promoter region of CYP2E1 are considered to affect the transcriptional activation of CYP2E1 gene [8]. The polymorphisms result in three genotypes, namely, wild-type homozygous (c1c1), heterozygous (c1c2), and variant homozygous (c2c2) genotypes.

Numerous studies on the possible association of CYP2E1 RsaI/PstI polymorphisms with gastric cancer risk have been conducted. However, the results are controversial. Whether CYP2E1 RsaI/PstI genetic variations can elevate the gastric cancer risk remains uncertain. Thus, in this study, we conducted a quantitative meta-analysis that included published data up to March 2012. This coverage increased the statistical power to determine accurately the relationship between CYP2E1 RsaI/PstI polymorphisms and gastric cancer risk.

## Materials and Methods

### 1 Literature Search Strategy

We carried out searches in Medline, EMBASE, OVID, Sciencedirect, and Chinese National Knowledge Infrastructure (CNKI) without a language limitation, covering all papers published up to Mar 2012. The following keywords were used: *Cytochrome P4502E1*, *CYP2E1*, *gastric*, *neoplasm*, *cancer*, *variation*, and *polymorphism*. All searched studies were retrieved and the bibliographies were further checked for other relevant publications. Review articles and the bibliographies of other relevant studies identified were hand searched to identify additional eligible studies.

**Figure 1:**
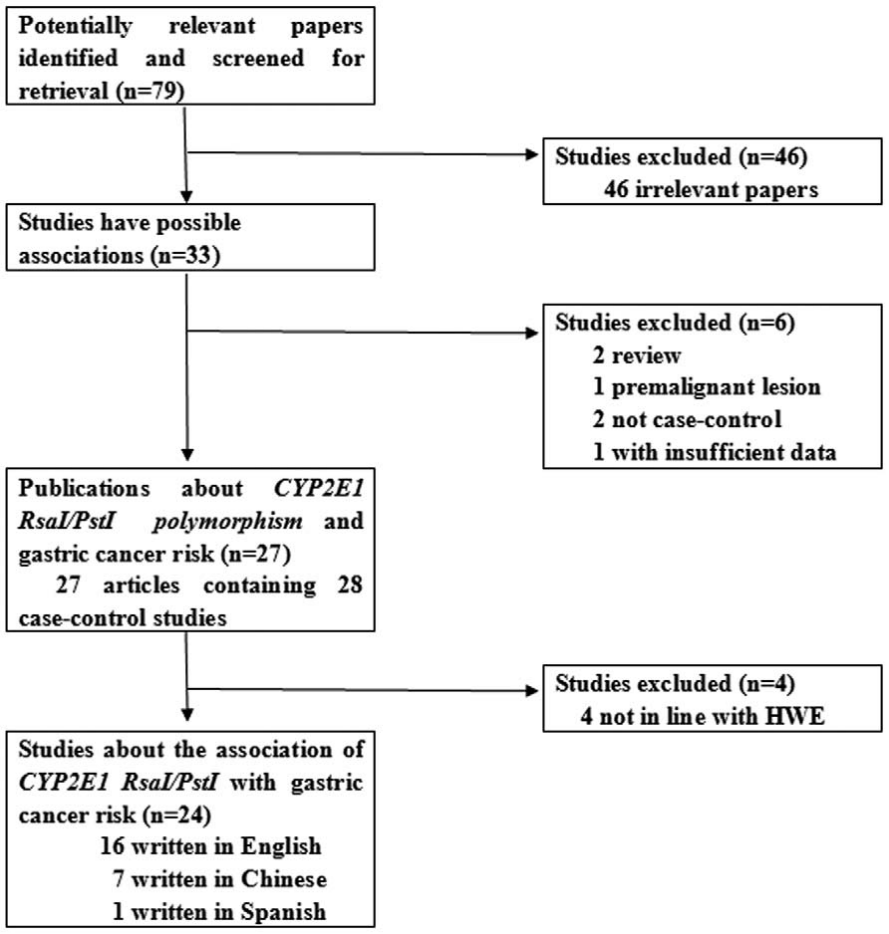
The flow diagram of included/excluded studies.

**Table 1:** Characteristics of studies included in the meta-analysis.

First Author	Publication Year	Number of Cases (male/female)	Number of Controls (male/female)	Type of controls	Median (or mean) age, (range) year (Cases/Controls)	Racial decent	Country
Kato	1995	150 (NA/NA)	203 (NA/NA)	Non-cancer patients with gastric disease (HB)	NA/NA	Asian	Japan
Kato	1996	82 (47/35)	151 (83/68)	Non-cancer controls (age-, gender-matched; HB)	61.8(33–84)/60.0(32–81)	Asian	Japan
Wang	1998	83 (50/33)	83 (50/33)	Healthy controls (age-, sex-matched; PB)	59.6(NA)/56.7(NA)	Asian	China
Nishimoto (Japanese)	2000	96 (60/36)	192 (120/72)	101 inpatients, 11 outpatients, 80 healthy volunteers (age-, gender-, ethnicity-, trimester of hospital admission-matched; HB)	65(37–89)/65(NA)	Asian	Brazil
Cai	2001	91 (77/14)	94 (82/12)	Healthy controls (age-, sex-matched; PB)	58.4(32–78)/58.2(34–79)	Asian	China
Qian	2001	306 (224/82)	164 (118/46)	Healthy controls (PB)	61.54(37–82)/61.46(32–87)	Asian	China
Gao	2002	98 (75/23)	196 (131/65)	Healthy controls (age-, sex-, ethnicity-matched; PB)	NA(40–81)/NA(40–81)	Asian	China
Ye	2002	56 (42/14)	56 (39/17)	Healthy controls (age-, sex-matched; PB)	57.6(22–79)/58.0(26–86)	Asian	China
Tsukino	2002	120 (82/38)	158 (109/49)	Healthy controls (age-, gender-matched; PB)	61.8(NA)/61.9(NA)	Asian	Japan
Wu	2002	356 (218/138)	278 (156/122)	Healthy controls (PB)	62.0(25–87)/61.6(22–86)	Asian	China
Zheng	2002	92 (74/18)	92 (74/18)	Healthy controls (age-matched; PB)	53.2(33–67)/53.5(35–68)	Asian	China
Park	2003	120 (77/43)	145 (89/56)	Non-cancer controls (age-, sex-matched; HB)	54.6(NA)/55.1(NA)	Asian	Korea
Zhou	2003	145 (113/32)	229 (155/74)	Healthy controls (age-matched; PB)	NA/NA	Asian	China
Suzuki	2004	146 (97/49)	177 (120/57)	Non-cancer controls (age-matched; HB)	62.4(30–84)/66.6(20–93)	Asian	Japan
Colombo	2004	100 (73/27)	150 (90/60)	Healthy control (age-, gender, ethnicity-matched; PB)	60(28–93)/54(20–93)	Mixed	Brazil
Gonzalez	2004	31 (25/6)	51 (33/18)	Non-cancer controls (HB)	60.71(32–78)/51.73(18–76)	Mixed	Costa Rica
Nan	2005	110 (70/40)	220 (140/80)	Non-cancer controls (age-, sex-matched; HB)	59.81(NA)/59.6(NA)	Asian	Korea
Wang	2005	48 (31/17)	48 (28/20)	Non-cancer controls (HB)	NA(50–70)/NA(55–72)	Asian	China
Agudo	2006	243 (NA/NA)	946 (NA/NA)	Non-cancer controls (age-, gender-, center-, blood collection date-matched; HB)	NA/NA	Caucasian	Ten European countries
Boccia	2007	107 (56/51)	254 (141/113)	Non-cancer controls (age-, gender-matched; HB)	66.4(NA)/64.0(NA)	Caucasian	Italy
Li	2007	41 (26/15)	41 (24/17)	Healthy controls (age-, sex-matched; PB)	52.8(40–71)/56.6(42–71)	Asian	China
Malik	2009	108 (90/18)	195 (139/56)	Healthy control (age-matched; PB)	55.91(NA)/57.98(NA)	Asian	India
Darazy	2011	13 (NA/NA)	70 (49/21)	Healthy control (age-, sex-matched; PB)	60.3(NA)/62.8(NA)	Mixed	Egypt
Kato	2011	464 (310/NA)	553 (322/231)	Non-cancer controls (age-, sex-matched; HB)	63.0(NA)/51.4(NA)	Asian	Japan

NA: not available;

PB: population-based;

HB: hospital-based.

### 2 Inclusion Criteria

The following criteria were used for the literature selection. First, the study should concern the association of CYP2E1 RsaI/PstI polymorphisms with gastric cancer risk. Second, the study must be observational (case-control or cohort). Third, the study must indicate the sample size, odds ratios (ORs), and their 95% confidence intervals (CIs), as well as the genetic distribution or the information that can help infer the results. After rigorous searching, we reviewed all papers based on the above criteria for further analysis.

**Table 2:** Distribution of CYP2E1 RsaI/PstI genotype among gastric cancer cases and controls included in the meta-analysis.

First Author	year	Genotyping method	Cases			Controls			HWE (control)	
			c2c2	c1c2	c1c1	c2c2	c1c2	c1c1	Chi-squre	P
Kato	1995	PCR-RFLP	6	54	90	14	69	120	0.867	>0.05
Kato	1996	PCR-RFLP	29 (a)	–	55	61 (a)	–	87	–	–
Wang	1998	PCR-RFLP	7	25	51	2	23	58	0.025	>0.05
Nishimoto (Japanese)	2000	PCR-RFLP	1	27	31	6	58	69	2.061	>0.05
Cai	2001	PCR-RFLP	6	27	58	1	22	71	0.243	>0.05
Qian	2001	PCR-RFLP	7	47	88	8	68	88	1.276	>0.05
Gao	2002	PCR-RFLP	9	31	58	13	62	121	1.641	>0.05
Ye	2002	PCR-RFLP	4	13	39	6	24	26	0.017	>0.05
Tsukino	2002	PCR-RFLP	7	42	71	12	58	88	0.317	>0.05
Wu	2002	PCR-RFLP	33	108	215	9	70	199	0.840	>0.05
Zheng	2002	PCR-RFLP	31 (a)	–	61	47 (a)	–	45	–	–
Park	2003	PCR-RFLP	7	33	80	3	48	94	1.235	>0.05
Zhou	2003	PCR-RFLP	15	45	85	14	75	140	0.840	>0.05
Suzuki	2004	PCR-RFLP	38 (a)	–	107	65 (a)	–	112	–	–
Colombo	2004	PCR-RFLP	0	11	89	0	16	134	0.476	>0.05
Gonzalez	2004	PCR-RFLP	5	15	31	0	11	20	1.442	>0.05
Nan	2005	PCR-RFLP	39 (a)	–	69	88 (a)	–	129	–	–
Wang	2005	PCR-RFLP	1	14	33	3	23	22	0.892	>0.05
Agudo	2006	PCR-RFLP	0	13	226	1	39	880	0.676	>0.05
Boccia	2007	PCR-RFLP	5 (a)	–	102	20 (a)	–	234	–	–
Li	2007	PCR-RFLP	6	10	25	8	16	17	1.328	>0.05
Malik	2009	PCR-RFLP	0	20	88	1	17	177	0.689	>0.05
Darazy	2011	PCR-RFLP	0	1	12	0	4	66	0.061	>0.05
Kato	2011	PCR-RFLP	186 (a)	–	280	213 (a)	–	340	–	–

(a): c2c2+ c1c2.

**Table 3:** Distribution of CYP2E1 RsaI/PstI genotype among ever-smokers and never-smokers bearing gastric cancers in the meta-analysis.

First Author	year	Cases		Controls	
		c2c2+c1c2	c1c1	c2c2+c1c2	c1c1
Ever smoking					
Cai	2001	23	37	11	23
Gao	2002	32	41	35	75
Zhou	2003	47	66	42	83
Agudo	2006	9	151	18	503
Boccia	2007	1	49	9	99
Never smoking					
Cai	2001	10	21	12	48
Gao	2002	8	17	37	44
Zhou	2003	12	19	45	54
Agudo	2006	4	79	22	403
Boccia	2007	4	53	11	135

**Table 4:** Distribution of CYP2E1 RsaI/PstI genotype among ever-drinkers and never-drinkers bearing gastric cancers in the meta-analysis.

First Author	year	Cases		Controls	
		c2c2+c1c2	c1c1	c2c2+c1c2	c1c1
Ever drinking					
Cai	2001	19	32	8	20
Gao	2002	5	9	9	13
Zhou	2003	23	33	22	33
Suzuki	2004	17	48	13	32
Boccia	2007	5	68	10	123
Never drinking					
Cai	2001	14	26	15	51
Gao	2002	35	49	66	108
Zhou	2003	36	49	66	107
Suzuki	2004	20	51	34	51
Boccia	2007	0	32	10	111

### 3 Data Extraction

Data were carefully extracted from all eligible publications by two of the authors **(Zhuo and Zhang)** independently in accordance with the aforementioned inclusion criteria. For conflicting evaluations, an agreement was reached after a discussion. When a consensus cannot be reached, another author was to be consulted to resolve the dispute, and then a final decision was made based on a majority of votes. The extracted information was inputted into a database.

### 4 Statistical Analysis

The ORs of CYP2E1 RsaI/PstI polymorphisms and gastric cancer risk were estimated for each study. The pooled ORs were determined for an allelic contrast model (c2 allele vs. c1 allele), a homozygote comparison model (c2c2 vs. c1c1), and a dominant model (c2c2+c1c2 vs. c1c1). To detect any possible sample size bias, the OR and its 95% CI for each study were plotted against the number of participants. The *I*-squared value was used as an index for the heterogeneity test [9], with values less than 25% indicating low, 25% to 50% indicating moderate, and greater than 50% indicating high heterogeneity. A chi-squared-based Q-statistic test was also performed to assess heterogeneity. If the *P* value for the Q-test was more than 0.1, ORs were pooled according to the fixed-effect model (Mantel-Haenszel) [10]; otherwise, the random-effect model (DerSimonian and Laird) was used [11]. The significance of the pooled ORs was determined by the Z-test. The Hardy-Weinberg equilibrium (HWE) was assessed by Fisher’s exact test. Publication bias was assessed by visual inspection of funnel plots [12] **,** in which the standard error of log (OR) of each study was plotted against its log (OR). An asymmetric plot indicated possible publication bias. The symmetry of the funnel plot was further evaluated by Egger’s linear regression test [13]. Statistical analysis was performed using the program STATA 11.0 software (Stata Corporation, Texas, USA).

**Table 5:** Main results of the pooled data in the meta-analysis.

	No. (cases/controls)			c2 vs c1								c2c2 vs c1c1					(c2c2+c1c2) vs c1c1			
				OR (95%CI)		P		P (Q-test)		*I* ^2^		OR (95%CI)		P	P (Q-test)	*I* ^2^	OR (95%CI)	P	P (Q-test)	*I* ^2^
Total	3022/4635			1.06 (0.88–1.28)		0.539		0.001		59.0%		1.23 (0.78–1.92)		0.370	0.034	43.2%	0.93 (0.79–1.10)	0.413	0.001	52.6%
Ethnicity																				
Asian	2512/3210			1.03 (0.83–1.28)	0.770			0.001		68.0%		1.18 (0.74–1.88)		0.489	0.023	48.2%	0.92 (0.76–1.11)	0.363	0.000	61.4%
Caucasian	346/1174			1.23 (0.65–2.31)	0.526			–		–		1.30 (0.05–31.91)		0.874	–	–	0.94 (0.44–2.00)	0.872	0.192	41.1%
Mixed	164/251			1.25 (0.73–2.15)	0.416			0.802		0.0%		7.16 (0.38–136.50)		0.191	–	–	1.11 (0.62–2.00)	0.729	0.963	0.0%
Sourceof controls																				
HB	1577/2829		0.95 (0.74–1.21)		0.653		0.262		22.8%		0.92 (0.37–2.33)		0.868		0.199	31.5%	0.89 (0.76–1.04)	0.153	0.361	8.7%
PB	1445/1806		1.12 (0.87–1.43)		0.375		0.001		65.7%		1.36 (0.81–2.27)		0.248		0.041	48.7%	1.00 (0.75–1.33)	0.995	0.000	66.0%
Smokingstatus																				
Ever smoking		456/898		–		–		–		–		–		–	–		1.39 (1.00–1.92)	0.049	0.481	0.0%
Never smoking		227/811		–		–		–		–		–		–	–		0.90 (0.58–1.39)	0.635	0.498	0.0%
Drinkingstatus																				
Ever drinking		259/283		–		–		–		–		–		–	–		1.02 (0.67–1.56)	0.934	0.933	0.0%
Never drinking		312/619		–		–		–		–		–		–	–		1.03 (0.76–1.39)	0.860	0.169	37.8%

PB: population-based;

HB: hospital-based.

**Figure 2:**
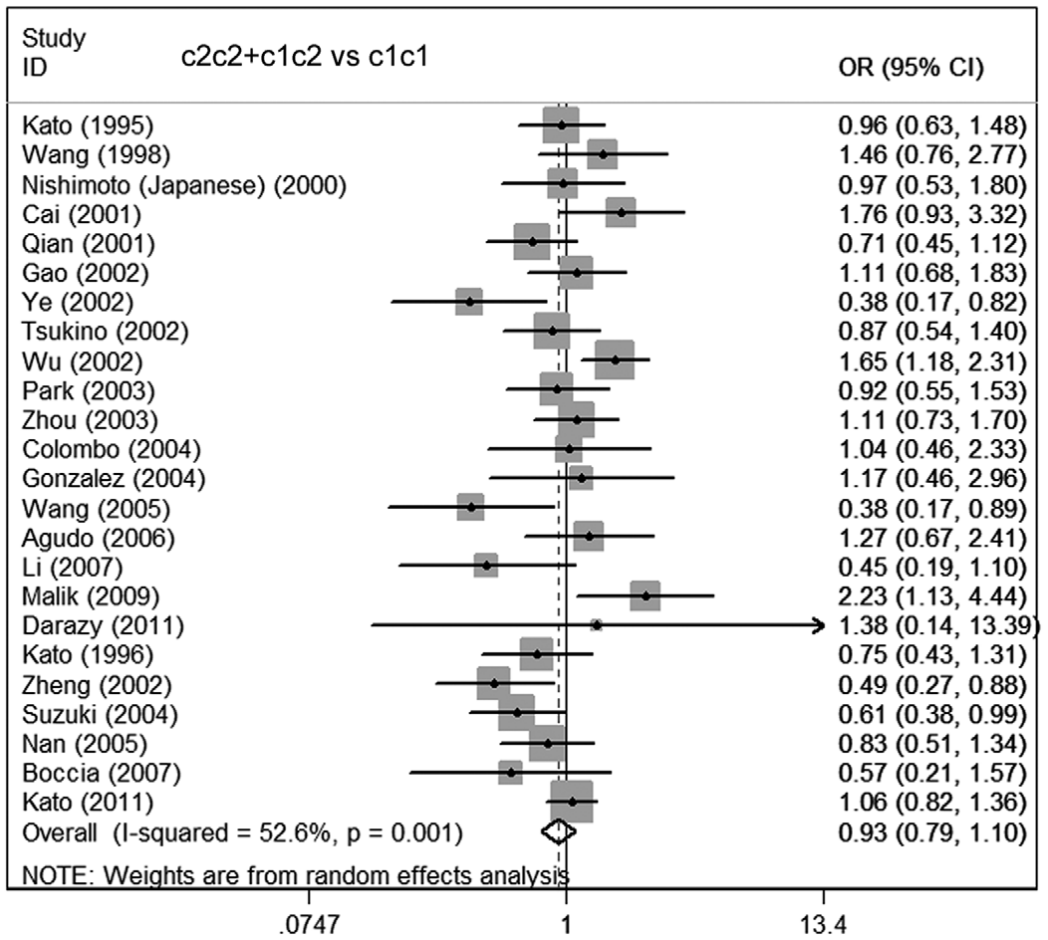
Meta-analysis for the association of gastric cancer risk with CYP2E1 RsaI/PstI polymorphism for the overall data (c2c2+c1c2 vs c1c1).

## Results

### 1 Study Characteristics

Relevant publications were retrieved and preliminarily screened. As shown in **Figure 1**, 79 publications were identified, among which 46 irrelevant papers were excluded. Thus, 33 publications were eligible. Two review articles [14], [15] and one paper on precancerous gastric lesions [16] were discarded. Two non-case-control studies [17], [18] and one study without detailed information [19] were also excluded. As a result, 27 publications containing 28 case-control studies were selected for data extraction and assessment. Notably, one study conducted in Brazil [20] involved two separate subgroups, namely, Brazilian and Japanese, respectively. Consequently, the data were extracted and considered as two solitary studies for analysis. Afterwards, three studies [21], [22], [23] and the mentioned Brazilian study [20] were further discarded because the genetic distributions of the controls significantly deviated from the HWE. Lastly, 24 case-control studies were included in the meta-analyses [20], [24], [25], [26], [27], [28], [29], [30], [31], [32], [33], [34], [35], [36], [37], [38], [39], [40], [41], [42], [43], [44], [45], [46].

Sixteen publications were written in English [20], [24], [25], [26], [27], [28], [29], [30], [31], [32], [33], [34], [36], [37], [38], [39], seven in Chinese [40], [41], [42], [43], [44], [45], [46] **,** and one in Spanish [35]. The relevant information is listed in **Table 1**. The first author, the number and characteristics of cases and controls for each study, and other necessary information are presented.

**Figure 3:**
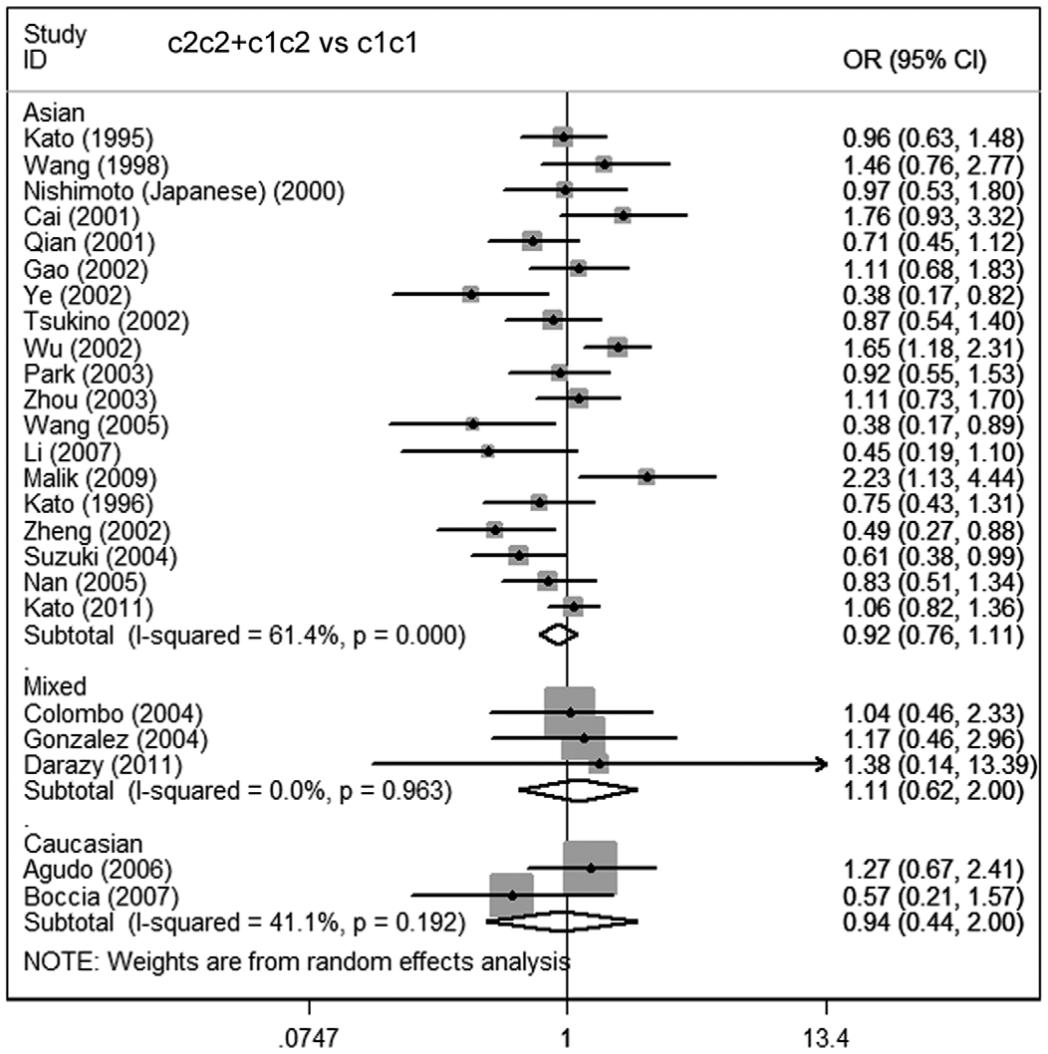
Meta-analysis for the association of gastric cancer risk with CYP2E1 RsaI/PstI polymorphism (c2c2+c1c2 vs c1c1; stratified by ethnicity).

**Figure 4:**
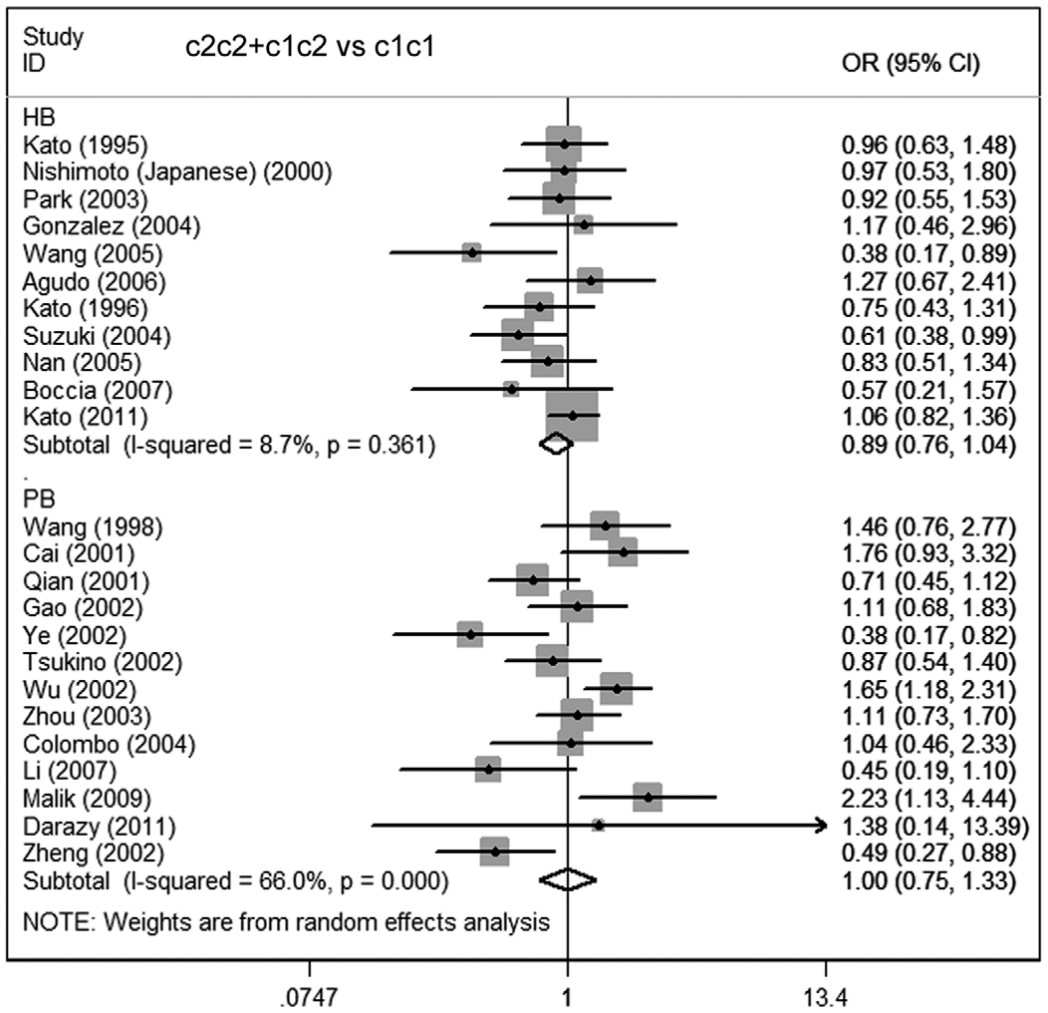
Meta-analysis for the association of gastric cancer risk with CYP2E1 RsaI/PstI polymorphism (c2c2+c1c2 vs c1c1; stratified by source of controls). PB: population-based; HB: hospital-based.

**Figure 5:**
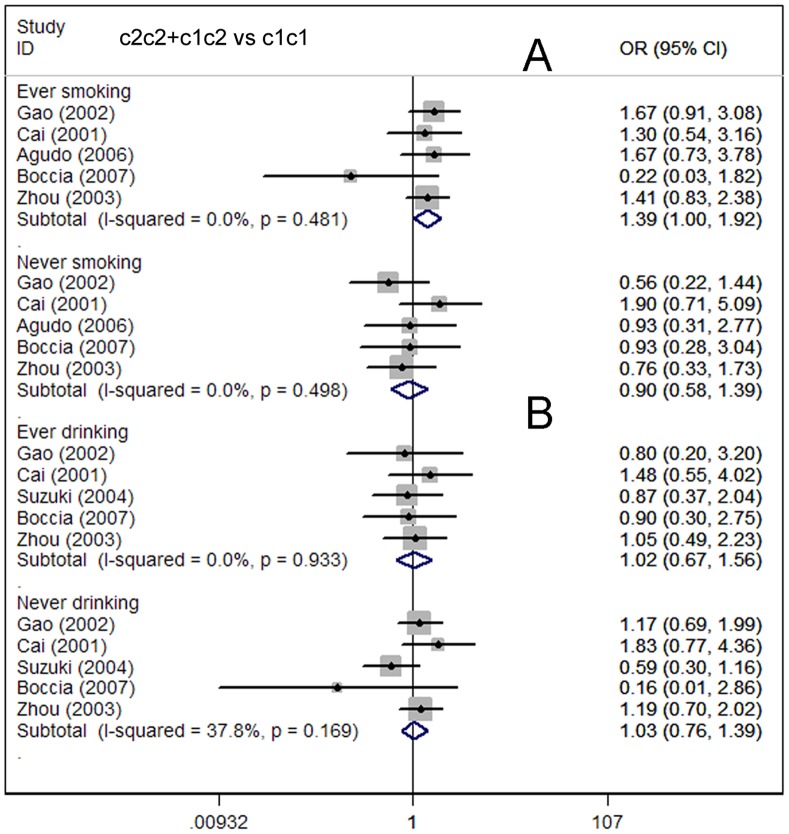
Meta-analysis for the association of gastric cancer risk with CYP2E1 RsaI/PstI polymorphism stratified by smoking status and alcohol consumption (c2c2+c1c2 vs c1c1; A: smoking status; B: drinking status).

**Figure 6:**
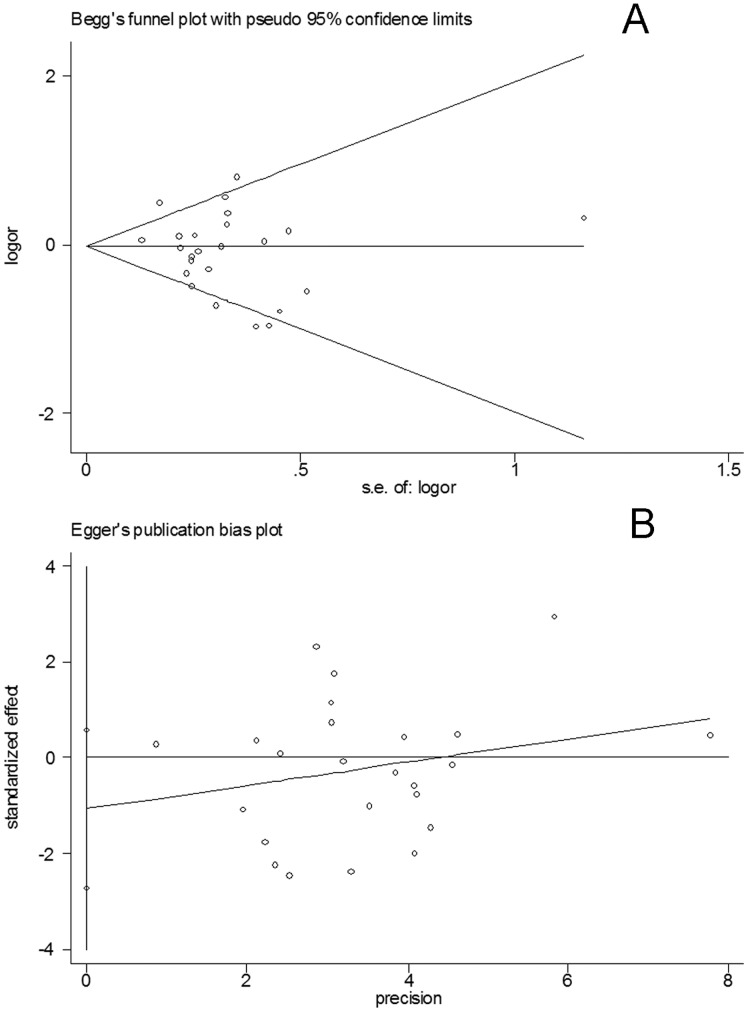
Publication bias test for the overall data (c2c2+c1c2 vs c1c1; A: Funnel plot; B: Egger’s linear regression test).

The selected articles included two groups of Caucasians [34], [36], nineteen of Asians [20], [24], [25], [26], [27], [28], [29], [30], [31], [33], [37], [39], [40], [41], [42], [43], [44], [45], [46], and three of mixed ethnicities [32], [35], [38].

The distributions of the CYP2E1 RsaI/PstI genotypes and the genotyping methods of the included studies are presented in **Table 2**. The genetic distributions of the control groups in all studies were consistent with the HWE. The genetic distributions of variant c2c2 and c1c2 in six included studies were combined as c2c2+c1c2 [25], [31], [33], [36], [39], [44]. The detailed genetic distributions were not available in the primary literature.

Data regarding smoking status were obtained from five studies [26], [27], [34], [36], [42] **(** **Table 3** **).** As for alcohol consumption, information was extracted from five studies [26], [27], [31], [36], [42] **(** **Table 4** **)**. The studies regarding smoking and drinking only provided the combined genetic distributions (c2c2+c1c2) for variant genotypes rather than the separate genotypes.

### 2 Test of Heterogeneity

As shown in **Table 5**, we analyzed the heterogeneity for the allelic contrast (c2 allele vs. c1 allele), homozygote comparison (c2c2 vs. c1c1), and dominant (c2c2+c1c2 vs. c1c1) models, respectively. Studies that provided the combined genetic distributions (c2c2+c1c2) rather than the separate genotypes were included only in the dominant model.

Marked heterogeneities for the overall data were found in three models (c2 vs. c1: *I* ^2^ = 59.0%; *P* = 0.001 for Q-test; c2c2 vs. c1c1: *I* ^2^ = 43.2%; *P* = 0.034 for Q-test; c2c2+c1c2 vs. c1c1: *I* ^2^ = 52.6%; *P* = 0.001 for Q-test), respectively. However, the subgroup analyses revealed reduced or removed heterogeneities in several subgroups.

### 3 Meta-analysis Results

The main results of the meta-analysis are listed in **Table 5**. For the overall data including 3022 cases and 4635 controls, the pooled ORs for the allelic contrast, homozygote comparison, and dominant models were 1.06 (95% CI = 0.88–1.28), 1.23 (95% CI = 0.78–1.92), and 0.93 (95% CI = 0.79–1.10), respectively. These results indicated that CYP2E1 RsaI/PstI variations may have little association with increased or decreased gastric carcinoma susceptibility **(** **Figure 2** **)**.

Considering the potential impact of the confounding factors on the overall results, we further performed subgroup analyses. In the primary literature, only the detailed information on ethnicity, source of controls, smoking and drinking status were sufficient for analysis. Hence, subgroup analyses on these issues were carried out. In the subgroup analysis on ethnicity, no significant association was found in the Asian, Caucasian, or mixed-ethnicity subgroups **(** **Figure 3** **)**. Similar results were observed in the subgroup analysis on the source of controls. No increased or decreased risk was found in the hospital- and population-based subgroups **(** **Figure 4** **)**. However, in the smoking status subgroups, a borderline increase in cancer risk was found among long-term smokers (OR = 1.39; 95% CI = 1.00–1.92; *P* = 0.481 for heterogeneity) but not among non-smokers (OR = 0.90; 95% CI = 0.58–1.39; *P* = 0.498 for heterogeneity) **(** **Figure 5A** **)**. This finding suggested that the interaction of CYP2E1 polymorphisms with cigarette smoking may slightly increase the gastric carcinoma susceptibility. In the subgroup analysis on alcohol consumption, no association was observed in long-term drinkers or non-drinkers **(** **Figure 5B** **).**

### 4 Sensitivity Analysis

When the effect models were changed, the significance of the overall data for the three models was not statistically altered (data not shown). One-way sensitivity analysis [47] was performed to evaluate the stability of the meta-analysis. The statistical significances of the overall results did not change when any single study was omitted (data not shown), indicating the stability of the results.

### 5 Bias Diagnostics

Funnel plots were created to assess possible publication biases. Then, Egger’s linear regression tests were performed to assess the symmetries of the plots. The funnel plots appeared to be symmetrical for the overall data **(** **Figure 6A** **)**. The results of the Egger’s tests also indicated the absence of publication biases **(** **Figure 6B** **)** (c2 vs. c1: *t* = −0.76, *P*>0.05; c2c2 vs. c1c1: *t* = −0.48, *P*>0.05; c2c2+c1c2 vs. c1c1: *t* = −1.35, *P*>0.05).

## Discussion

The results showed that CYP2E1 RsaI/PstI polymorphisms may not be correlated with gastric cancer risk. Similar results were found in the subgroups stratified by ethnicity, source of controls, and drinking status. However, in the subgroup analysis on smoking status, the data indicated increased gastric cancer risk in long-term smokers.

A previous meta-analysis by Boccia et al. [48] that included 13 studies prior to year 2006 shows increased gastric cancer risk in Asians. The study also indicated that the interactions of CYP2E1 polymorphism with smoking have little association with gastric cancer risk, in contrast with the present, updated meta-analysis. In the present study, 24 case-control studies involving 3022 cases and 4635 controls were selected. In our primary analyses, 28 case-control studies were selected. However, unstable results for the overall data were found when a sensitivity analysis was performed. Studies whose genetic distributions of controls significantly deviate from the HWE were discarded, considering that the deviation may contribute to bias [49]. As expected, stable results were obtained; thus, the credibility and robustness of the results were significantly increased.

In the subgroup analysis on ethnicity, no significant association was found among Asians, Caucasians, and mixed-ethnicity subgroups, in line with the overall data. Ethnic variations in various genes among different ethnicities may influence gastric cancer susceptibility [50], [51]. CYP2E1 variations differ among various ethnicities [52]. Thus, CYP2E1 variations may exert different influences on gastric cancer risk among different races. Nevertheless, the data of the present study suggested that the interactions of CYP2E1 RsaI/PstI polymorphisms with ethnic variations may exert little influence on gastric cancer susceptibility. In the present meta-analysis, only two groups of Caucasians were obtained. The results may be due to chance because the limited number of included studies and small sample sizes may give rise to insufficient statistical power to assess a minor effect. Thus, the results should be interpreted with caution. Further investigations with large sample sizes regarding Caucasians are needed to clarify the possible effects of CYP2E1 ethnic variations on gastric cancer risk.

In the subgroup analysis on the source of controls, significantly increased and decreased gastric cancer risks were not observed in the hospital- and population-based subgroups. Hospital-based controls may not be always truly on behalf of the general population, and may thus underestimate the gastric cancer risk. Therefore, selection bias may exist. Further studies using proper controls with strict matching criteria and large sample sizes are important to reduce such selection biases. However, the data of the present meta-analysis indicated that the selection biases hardly affected the results.

Smoking is an important established risk factor for gastric cancer. The data of our meta-analysis showed a borderline increase in gastric cancer risk among long-term smokers, in contrast with the results of Boccia et al. [48]. Tobacco smoke contains many carcinogens, such as benzopyrene and nitrosamine. These compounds are metabolized by phase-I enzymes including CYP family enzymes, and converted to inactive metabolites by the phase-II enzymes. Previous reports showed that mutant alleles of CYP2E1 have increased transcriptional activity [53]. Cigarette smoking can significantly accelerate chlorzoxazone metabolism and enhance the activity of CYP2E1 [54], [55], which may markedly activate a number of carcinogens and thereby result in increased gastric carcinoma risk among long-term smokers. This finding may explain the ability of CYP2E1 polymorphism to increase the cancer risk among long-term smokers. However, only five of the included studies provided sufficient data on smoking status with relatively limited sample sizes. Therefore, the data may underestimate the gastric carcinoma risk and should be interpreted with caution.

In the subgroup analysis on alcohol consumption, no increased cancer risk was found in long-term drinkers or non-drinkers. CYP2E1 can metabolize and activate many toxicological substrates, including ethanol, to become more reactive, toxic products. Thus, its levels may be elevated after chronic or acute alcohol treatment [56]. Therefore, the effect of the interactions between CYP2E1 polymorphism and alcohol consumption on cancer risk should be noted. A recent meta-analysis on hepatocellular cancer suggested that Pst I/Rsa polymorphisms can elevate cancer susceptibility among long-term drinkers [57]. However, only five studies with limited sample sizes concerning drinking status were included in the present study, with possible biases generated. Further investigations on the effect of the interactions of CYP2E1 polymorphism and drinking on gastric cancer are required to address this controversy.

In the present meta-analysis, evident between-study heterogeneities for the overall data were observed in the three genetic models; thus, random-effect models were utilized. In the subgroup analyses, removed heterogeneities were also found in the subgroup analysis on Caucasian and mixed ethnicities, as well as on hospital-based controls. Nevertheless, significant heterogeneities were still found in the subgroup analysis on Asians and population-based controls. The data suggested that the heterogeneities may be multifactorial. In addition to the ethnicity and source of controls, other factors such as age, gender, and histological types may also contribute to the heterogeneities.

Publication bias is an important factor that should be considered in a meta-analysis. We utilized funnel plots to evaluate the possible publication biases. Then, Egger’s linear regression test was performed to evaluate their symmetries. The results did not suggest evident biases, which indicated the robustness and credibility of the results.

Several limitations should be addressed. First, in this meta-analysis, the primary articles only provided data about Caucasians, Asians, and mixed ethnicities. Most of the studies concerned Asians and only two studies concerned Caucasians. Data regarding other ethnicities, such as African, were not available. Second, subgroup analyses on age, gender, histological types, and other factors (such as *H. pylori* infection, an important risk factor for gastric cancer) were not conducted in the present study because relevant data were not available in the primary literature. Third, the sample sizes for a proportion of included studies were relatively small; the matching criteria for the cases and controls were also not strict. Thus, bias may exist. Among the included studies, other genes such as GSTM1 and NAT2 were of concern in several papers. However, the interactions between CYP2E1 RsaI/PstI and other gene polymorphisms can be found in only one of the included studies [31]. Therefore, gene–gene interactions cannot be performed as a subgroup analysis because of the insufficient information. Further investigations with larger sample sizes and strict matching criteria in view of more confounding factors are needed to address the possible associations.

In summary, although the overall data failed to reveal a significant association of CYP2E1 RsaI/PstI polymorphism with gastric cancer risk, the subgroup analyses indicated that the variant c2 allele of CYP2E1 RsaI/PstI may modify gastric carcinoma susceptibility among individuals who have a smoking history.
